# Determinants of village doctors’ job satisfaction under China’s health sector reform: a cross-sectional mixed methods study

**DOI:** 10.1186/s12939-017-0560-8

**Published:** 2017-04-18

**Authors:** Tongtong Li, Trudy Lei, Fiona Sun, Zheng Xie

**Affiliations:** 1 School of Public Health, Peking University, 38 Xueyuan Road, Haidian District, Beijing, 10091 People’s Republic of China; 2 School of Public Health of Columbia University, New York, USA; 3 London School of Hygiene and Tropical Medicine, London, UK

**Keywords:** Village doctors, Job satisfaction, Rural health workforce

## Abstract

**Background:**

To strengthen rural health workforce, the Chinese government has launched a series of policies to promote the job satisfaction of village doctors since the health sector reform. The purpose of this mixed-method study is to describe village doctors’ job satisfaction under the context of health sector reform and investigate the associated factors.

**Methods:**

Data was obtained from a survey of village doctors across three Chinese provinces in 2014. Using a multistage sampling process, quantitative data was collected from village doctors through the self-administered questionnaire and analyzed by multilevel logistic regression models. Qualitative data was collected through face-to-face semi-structured interviews on both village doctors and health managers. Theoretical coding was then conducted to analyze qualitative data.

**Results:**

Among the 1221 respondents, 48.6% felt satisfied with their job. Older village doctors with less of a workload and under high-level integrated management were more likely to feel satisfied with their job. Village doctors who earned the top level of monthly income felt more satisfied, while on the county level, those who lived in counties with the highest GDP felt less satisfied. However, enrollment in a pension plan showed no significant difference in regards to village doctors’ job satisfaction.

Among 34 participants of qualitative interviews, most believed that age, income, and integrated management had a positive influence on the job satisfaction, while pension plan and basic public health care policies exhibited negative effects. Also, the increasing in availability of healthcare and health resources along with local economic development had negative effects on village doctors’ job satisfaction.

**Conclusion:**

Village doctors’ job satisfaction was quite low in regards to several determinants including age, income, workload, enrollment in a pension plan, integrated management, and county economic and medical availability development.

## Background

China is experiencing critical challenges in the rural health system related to the low job satisfaction of village doctors [1]. These doctors, formerly called “barefoot doctors”, serve as the bottom-tier of the Chinese rural health system. Many of them were originally employed as the primary rural health workforce in the 1960s, and their position in serving rural areas is still integral to basic rural healthcare provision in China nowadays [2]. However, since the economic reform in the early 1980s, the collapse of rural collective economy and the privatization of healthcare have led to a dramatic decline in rural healthcare funding [3]. Economic hardship has forced village doctors to shift their focus to fee-for-service medical activities and profits from a markup on prescribed and dispensed drugs [4, 5]. Also, the government did not provide any support to village doctors through the health system changes [6], leaving them out of the governmental system. These changes left village doctors less satisfied with their job.

Low job satisfaction can result in low performance and a brain drain of the health workforce, threatening the sustainability of the entire healthcare system [7, 8]. Primary health workers, who feel unsatisfied with their job, lack the enthusiasm and willingness to make an effort for high-level service provision, thereby indirectly working against health promotion [9, 10]. Additionally, with the Chinese economic development, there are more and more job options for primary health workers. Those who are underpaid and feel a lack of respect tend to switch jobs rather than remain in the bottom-tier of the healthcare system. This problematic situation in China also has policy implications for many developing countries undergoing urbanization and industrialization.

Fortunately, China’s central government initiated the Health Sector Reform in 2009, aiming to provide safe, effective, convenient, and affordable basic health care services for the 1.3 billion people living in both rural and urban areas [11]. Since then, more attention has been paid to the rural health system through implementation of a series of strategies for village-level health workforce strengthening. There are three policies that directly affect village doctors’ job satisfaction, namely provision of basic public health services, integrated management, and new pension scheme. The new policy encourages a shift of focus for village doctors from provision of basic medical care towards basic public health services [12] which include 11 items such as health records creation for every resident, health education, immunization, and chronic disease management forth [13]. The policy regarding integrated management requires upper-level health organizations, for instance, township health centers (THCs), to begin managing village clinics and overseeing medicine, personnel, finance, facilities, routine work, etc., thus allowing village doctors to focus on providing care rather than administrative tasks [14]. Lastly, the central government also launched a new pension plan providing basic social security and financial support post-retirement [15, 16], the New Rural Pension Program (NRPP), covering most rural residents in China. Though the NRPP was not exclusively for village doctors, they were encouraged to enroll as well, and thus it became the most widely enrolled-in pension scheme for village doctors [17]. This study focused on these three reformed policies and related factors affecting job satisfaction under such situation.

Has job satisfaction of the village doctors changed with the modification of their working conditions after the Reform? Few studies have answered this question. The only research on this topic was carried out in one province and suggested that the job satisfaction of village doctors was low [18]. However, it did not discuss the relevant policy factors and how they affect job satisfaction and using data from only one province ignores the varied economic status and health reform processes among different areas of China [19–21]. The purpose of this mixed-method study is to describe village doctors’ job satisfaction under the context of health sector reform and investigate the associated factors, using a survey data from three provinces.

## Methods

**Fig. 1:**
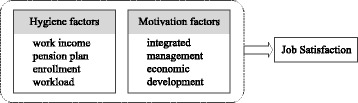
Theoretical model

We borrowed Herzberg’s Two-Factor Theory of motivation [22] as theoretical foundation to construct motivation factors of village doctors’ job satisfaction. According to the theory, two categories of motivation factors contribute to job satisfaction, namely hygiene factors and motivation factors. Hygiene factors cause dissatisfaction if not present which mainly include working conditions. In this study we defined income, pension plan enrollment, and workload as hygiene factors. Motivation factors are those relating to individual’s achievement, self-recognition and growth. Here we grouped integrated management and economic development into motivation factors (Fig. 1).

Based on the theoretical model, a cross-sectional mix methods study was designed. Questionnaire for quantitative survey and topic guide for qualitative survey were developed.

### Quantitative data

#### Sampling size

As prior studies showed [23], village doctors’ job satisfaction (p) is about 60%, with α at 0.05 (two side test) and a permissible error (d) of 0.1 × p, yielding a sample size of 267 by (1):

$$ \mathrm{n}=\frac{{\mathrm{z}}_{\propto}^2\times \mathrm{pq}}{{\mathrm{d}}^2} $$

Considering a 1.5 times group correction, a sample size of 400participants was calculated to obtain accurate estimates for the job satisfaction of village doctors in each province, totalling 1200 participants.

#### Data collection

**Fig. 2:**
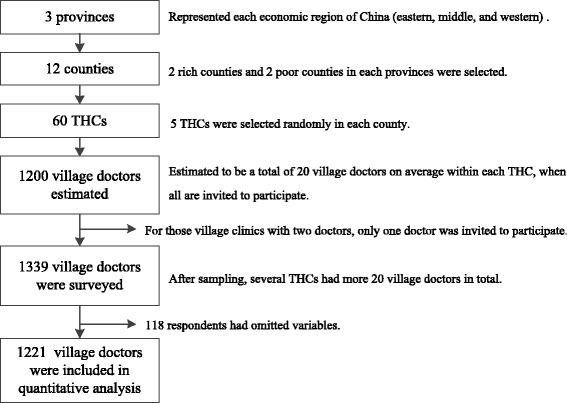
Flow chart for the sampling

Multi-stage sampling was conducted for the questionnaire survey in April 2014. Three provinces, Shandong, Guangxi, and Shaanxi, were selected randomly to represent different economic region of China (eastern, middle, and western). Within each province, counties were designated as “poor” or “wealthy” based on available socioeconomic status data [24], and two counties from each category were randomly selected. Five THCs were sampled in each county randomly. All village clinics under those five THCs were informed and encouraged to participate in the survey by managers of THCs. Generally each THC governed 20 village clinics, and each clinic has one or two doctors. The eligibility criteria was: (1) having worked in the clinics for at least six months, (2) be willing to attend the survey, (3) if a clinic had two doctors, only one was picked to participate. Each participant completed the survey independently. The study obtained a final sample size of 1339, while 118 respondents did not finish the questionnaire as required. Thus a final sample of 1221village doctors was included in the quantitative analyses. Participants came from 12 county-level units with 100–140 village doctors per county (Fig. 2).

#### Data measures

Job satisfaction of village doctors was measured by asking “Are you satisfied with your work?”, with responses of “very satisfied”, “satisfied”, “not satisfied” and “extremely not satisfied”. The answers “very satisfied” and “satisfied” were coded to be satisfied (coded 1); while “not satisfied” and “extremely not satisfied” were not satisfied (coded 0).

Control variables included age (20–39, 40–59, and 60 or over), gender (male, female), and education level (junior high school or less, secondary school, more than secondary school). Participants’ average monthly income ranged from 0 to 4000 CNY, separated equally into low, average, and high. Enrollment in a pension plan was measured by the question “Which kind of pension plan do you have?” with responses of “None”, “NRPP”, and “others”. Workload was measured by the question “How many hours do you work a day on average?” with a possible response ranging from 1 to 24 h. Then, a binary variable was formulated for low and high levels of workload using a break point at 10 h per day. Clinic participation in integrated management was measured by three separate questions, “Do THCs manage the finances/personnel salary/medical drugs of your village clinic?” Responses for each were recorded on a three-point scale: “total”, “partial”, and “none”, and given a value of 2 for “total”, 1 for “partial,” and 0 for ‘none’ with the sum score ranging from 0 to 6. Village clinics which scored 0, 1, 2, or 3, were considered as low level of integrated management (coded 0), and village clinics with scores of 4, 5, or 6 were considered high level (coded 1). Additionally, gross domestic product (GDP) in 2013 was also included as a county-level variable in order to account for economic development.

#### Data analysis

Data analyses were conducted using STATA version 11.0. Descriptive statistics were used to describe the characteristics of the study population. Chi-square tests were conducted to determine the differences among individuals’ varied satisfaction level. To model the effects of individual and county level variables on job satisfaction, data was fitted using a logistic regression with village doctors’ reported job satisfaction as the outcome. Odds ratios (ORs) and job satisfaction were analyzed by multilevel logistic regression models, adjusting both individual and county level variables as fixed effects and allowing for heterogeneity between counties. A series of three models was performed with Model 1 as a null model containing no explanatory variables. Intra-class correlation (ICC) coefficient was computed to examine the necessity of fitting multilevel models. Model 2 included all the variables at individual level. Model 3 added county level variables into Model 2.

### Qualitative data

#### Data collection

To reveal the crucial influencing factors of village doctors’ job satisfaction, after the initial quantitative phase that focused on village doctors only, we used qualitative methods with both village doctors and health managers using grounded theory. Face-to-face semi-structured interviews were conducted in this phase to enable flexible and in-depth exploration of this issue immediately after we finished the questionnaire survey with village doctors in April 2014. All the interviewers were experienced researchers and have been trained in qualitative research methodologies. They all gained PhD, M.D., or master degrees in public health or sociology. Two males and five females including two authors (TTL and ZX) composed this interviewer group. To be eligible, interviewees needed to be either village doctors who worked in the 12 chosen counties for more than six months or health managers who were responsible for village doctor issues. They were recruited through purposive sampling after the questionnaire survey, with consideration of gender, age, geographic location, and levels of seniority to include a representative range of opinions about village doctors’ job satisfaction. Sequential process was conducted until additional interviews did not provide further insights. Initially, 38 village doctors and health mangers were invited, while 2 village doctors and 2 health managers were not available during the research period. Thus, this phase had 34 participants, including 21 village doctors and 13 managers (See Appendix 1 for basic demographic information about interviewees).

The interviewers did not have any prior relationship with the participants and had no assumptions and personal interests during their participation in the study. Before the interviews started, aims of the study and how they were selected were introduced to participants. Interviews were conducted in an individual meeting room without any non-participants for privacy and quiet, as well as to ensure comfort of the participants. Each interview lasted between 30 and 60 min without repeating. Field notes and audio records were taken and transcribed verbatim by a professional freelance transcriptionist. No identifiable information was gathered and data was stored in locked files and treated with strict confidentiality.

#### Data analysis

Theoretical coding was conducted using NVivo 12.0. Through the initial abstraction and comparison, an early coding framework was developed by the research group. Through the coding process, more substantive codes that showed the relationships between codes were created. Coding consistency was continually checked and revised by reviewing transcripts, generating a final coding framework (Table 1). To fully illustrate the breadth of data, quotations were selected from the transcriptions by two researchers with consideration of inter-rater reliability (examples in results section). Member checking by inviting participants to review the quotations and results were conducted during the interview process and also at the conclusion of the study for respondent validation.

**Table 1:** Coding framework from the data

Factors influencing village doctors’ job satisfaction	
• Age • Income status • Pension plan • Workload • Integrated management • Health availability development	

## Results

Results of this mixed-method study are presented below. Quantitative data on overall job satisfaction of village doctors is presented and associated factors are explored first. Qualitative findings are then used to analyze these factors more deeply.

### Descriptive statistics of the sample

The majority of respondents were male (75.8%), with a secondary school degree (67.7%), and enrolled in NRPP (67.7%). Almost half were between 40 and 59 years old (53.5%). While 564 (46.2%) village doctors reported a high workload, 650 (53.2%) answered they worked in a village clinic with high-level integrated management (Table 2).

**Table 2:** Descriptive statistics

Variable	Number	Percentage (%)
Individual level (level-1, *n*=1221)		
Gender		
Male	925	75.8
Female	296	24.2
Age (years)		
20–39	356	29.2
40–59	653	53.5
60 +	212	17.4
Education		
≤Junior high school	153	12.5
Secondary school	827	67.7
>Secondary school	241	19.7
Average monthly income (CNY)		
Low	412	33.7
Ordinary	401	32.8
High	408	33.4
Pension plan		
None	316	25.9
NRPP	826	67.7
Others	79	6.5
Workload		
Low	657	53.8
High	564	46.2
Integrated Management		
Low	571	46.8
High	650	53.2
County level (level-2, *n*=12)		
GDP per capita		
Low	4	33.3
Average	4	33.3
High	4	33.3

### Differences in individual-level characteristics of village doctors’ job satisfaction

Among 1221 participants, 594 (48.6%) village doctors felt satisfied with their job at the high level (Table 3). More specifically, older village doctors, as well as those with lower education and high workload, felt more satisfied with their job (*P*<0.001). Monthly income and enrollment in a pension plan did not show any significant differences. To make the data distribution more clearly, we also calculated the relationship between varied variables (See Appendix 2).

**Table 3:** Differences in individual-level characteristics of village doctors’ job satisfaction (*N*=1221)

Variables	Low satisfaction (%)	High satisfaction (%)	*P* value^a^
Overall	627 (51.4)	594 (48.6)	
Gender			0.504
Male	480 (51.9)	445 (48.1)	
Female	147 (49.7)	149 (50.3)	
Age (years)			<0.001
20–39	211 (59.3)	145 (40.7)	
40–59	343 (52.5)	310 (47.5)	
60 +	73 (34.4)	139 (65.6)	
Education			<0.001
≤Junior high school	43 (28.1)	110 (71.9)	
Secondary school	443 (53.6)	384 (46.4)	
>Secondary school	141 (58.5)	100 (41.5)	
Average monthly income (CNY)			0.158
Low	208 (50.5)	204 (49.5)	
Ordinary	221 (55.1)	180 (44.9)	
High	198 (48.5)	210 (51.5)	
Pension plan			0.648
None	156 (49.4)	160 (50.6)	
NRPP	428 (51.8)	398 (48.2)	
Others	43 (54.4)	36 (45.6)	
Workload			<0.001
Low	261 (39.7)	396 (60.3)	
High	366 (64.9)	198 (35.1)	
Integrated Management			<0.313
Low	302 (52.9)	269 (47.1)	
High	325 (50.0)	325 (50.0)	

^a^ *P* value by chi-square test

### Multilevel logistic regression estimating village doctors’ job satisfaction

Table 4 shows the results of multilevel logistic regression analysis models testing the individual- and county-level factors. In Model 1, excluding explanatory variables, 18.2% of the variance in village doctors’ satisfaction came from the county-level (ICC coefficient = 0.182, *P*<0.001).

**Table 4:** Multilevel logistic regression estimates and variance components of village doctors’ job satisfaction

Variables	Model 2			Model 3		
	OR	*P* value	95% C.I.	OR	*P* value	95% C.I.
Individual level						
Gender						
Male (ref)						
Female	1.189	0.276	0.871, 1.624	1.194	0.265	0.874, 1.631
Age (years)						
20–39 (ref)						
40–59	1.518	0.007	1.123, 2.053	1.516	0.007	1.121, 2.050
60 +	3.434	<0.001	2.164, 5.447	3.427	<0.001	2.160, 5.436
Education						
≤ Junior high school (ref)						
Secondary school	0.591	0.024	0.375, 0.933	0.591	0.024	0.375, 0.932
> Secondary degree	0.703	0.202	0.409, 1.208	0.699	0.194	0.407, 1.200
Average monthly income						
Low (ref)						
Average	1.056	0.747	0.758, 1.471	1.051	0.767	0.755, 1.465
High	1.793	0.001	1.280, 2.512	1.767	0.001	1.261, 2.477
Pension plan						
None (ref)						
NRPP	0.796	0.436	0.448, 1.413	0.798	0.442	0.450, 1.418
Others	1.127	0.465	0.818, 1.551	1.111	0.519	0.807, 1.531
Workload						
Low (ref)						
High	0.519	<0.001	0.391, 0.689	0.514	<0.001	0.387, 0.683
Integrated Management						
Low (ref)						
High	1.455	0.020	1.061, 1.997	1.448	0.021	1.057, 1.983
County level						
GDP						
Low (ref)						
Average				0.438	0.136	0.148, 1.295
High				0.328	0.042	0.112, 0.960

In Model 2, older village doctors (OR=1.518, 95% CI: 1.123–2.053 and OR=3.434, 95% CI: 2.164–5.447) and village doctors who had low workloads (OR=0.519, 95% CI: 0.391–0.689) as well as working in high-level integrated management village clinics (OR=1.455, 95% CI: 1.061–1.997), were more likely to feel satisfied. Meanwhile, village doctors who had secondary school degree were less satisfied than those who had junior high school or lower degree (OR=0.024, 95% CI: 0.375–0.933). On the other hand, the varied pension plans that village doctors were enrolled in did not increase the probability of high satisfaction. The results were fairly constant even after including the county-level variable (Model 3).

In Model 3, village doctors who earned high monthly income felt more satisfied than those who earned low monthly income (OR=1.767, 95% CI: 1.261–2.477). No significant difference was found between low and average income groups. However, those practicing in counties with high GDP felt less satisfied (OR=0.328, 95% CI: 0.112–0.960), meanwhile there was no significant difference in counties with average and low GDP.

### Perceptions of factors influencing job satisfaction

#### Age

Level of job satisfaction varied by the age of village doctors. Most senior village doctors felt satisfied with their job, since they had longer careers, gained more trusted by the villagers, and carried out their work more smoothly. Middle age village doctors had encountered resistance in the beginning of their work, but with the increasing of their career life, this dilemma gradually resolved. However, many junior village doctors had high job expectations and believed being village doctors as a career could not meet their aspirations, which decreased their job satisfaction.

- “Although there have been many changes along with rapid development, patients still look for me when they get sick because of my reputation. All their family members know me and come to me for help.” (Participant 107, age 60+, village doctor)
- “People hardly knew me when I just came back home for the job in 1998. At that time, patients didn’t know of my abilities. Everything was difficult. It got better several years later, as I worked longer. Everything became easier than the very beginning.” (Participant 103, age 40–59, village doctor)
- “Nowadays it’s not just about being a village doctor, even being a doctor at a town hospital wouldn’t attract me much. It’s too risky and there’re too many hospital violators. We wouldn’t have become a village doctor if our moms didn’t force us to. If I didn’t compromise and become a village doctor, I could have been a millionaire now.” (Participant 114, age 20–39, village doctor)

#### Income status

Most village doctors thought their income was quite low, which influenced their job satisfaction significantly. Nowadays, through working in nearby cities as migrant workers or engaging in fruit and vegetable cultivation in local areas, many villagers usually could earn a comfortable living. Being a village doctors had no income advantage compared to the average income status in their villages. Some health managers also believed that several village doctors did not earn enough even for their basic life material needs.

- “I am really willing to be a village doctor; it’s a good job, you know. However, the income is too low to subsist on. I must earn what I need for living.” (Participant 304, village doctor)
- “Now there are more and more people breeding silkworms. They even earn more than us (village doctors).” (Participant 116, village doctor)
- “Village doctors’ incomes are low. A village here, only 500 residents live in. If a village doctor works there, he/she could not earn a living… Certainly, there are some other villages in relatively better situations.” (Participant 303, health manager)

#### Pension plan

Although there were several newly launched national policies focused on the pension problem of village doctors, some village doctors were reluctant to purchase NRPP. As health professionals working at township hospital and above had better government provided pension plans available to them, many village doctors felt disrespected that they were not treated the similarly. Health managers confirmed that NRPP covered a large varied population without considering village doctor’s professional value and thus, did not improve their job satisfaction.

- “Although there is NRPP as an option, it’s only enough for supplies. It’s too menial for a village doctor. Nowadays, village doctors are treated the same as normal villagers, with the same type of pension plan available for purchase.” (Participant 102, village doctor)
- “I think we deserve the same pension plan as those who work in THCs, because we are doing the same work everyday. Sometimes, we work even harder than them. It is only reasonable that we get the same pension plan.” (Participant 307, village doctor)
- “If village doctors work as one part of the regular health care system, they may feel satisfied with their jobs, as they are making a contribution. However, they have to purchase the same pension plan as normal villagers do. How can they accept that?”(Participant 101, health manager)

#### Workload

Village doctors not only provided basic medical services, but also offered basic public health care services to the entire community, which was increased since the Health Sector Reform in 2009. Due to the scattered locations of rural households, inconvenient transportation, and irregular working times of villagers, it took lots of time and energy for village doctors to offer basic public health care services in each household, decreasing their job satisfaction. As for this issue, health managers also reflected that there were difficulties on basic public health care services providing in rural areas, as it increasing village doctors’ workload significantly.

- “Too much workload now. I am in charge of only one village, with about 1500 residents. However, they live dispersedly. One is here, while another is quite far away. I run around all day long, but still can only offer public health services for several households.” (Participant 205, village doctor)
- “We walk around to offer public health care services, walking far lengths everyday. For example, a person inquires for us to come to his/her household. Sometimes when I go to their home, they go outside to work, and then I have to leave without any outcome. When I return the next day, he/she may still not be at home. In such situations, I sometimes cannot find the person after even ten visits. It’s so exhausting! Additionally, roads in rural areas are bad. I always feel quite tired everyday.” (Participant 105, village doctor)
- “Nowadays, workload of village doctors is quite heavy without question. Before 2009, village doctors worked privately, just sold drugs or did something like that. They probably stayed at their clinics in the mornings and did some farming work or run small business in the afternoons. But now, we require them to provide basic public health care services, and we evaluate their work by the amount of services they provide, such as how many chronic diseases follow-up finished and how many families have been informed about children vaccination. Village doctors need to offer these services door-to-door. It is indeed a substantial task, and village doctors are not truly willing to do this work.” (Participant 202, health manager)

#### Integrated management

Most village doctors thought integrated management differentiated them from private drug salesmen and made their work more formal and legal. Thus, they felt more respected and more satisfied. Meanwhile, health mangers reflected that THCs would manage village doctors as their own health workforce under the integrated management, which helped village doctors provide more consistency in medical service and promoted their job satisfaction in the long-term.

- “If you just sell medicine at home, people won’t respect you. They think you sell drugs for a living. With the integrated management system, everything is more official and proper. Wherever we go, people respect us, just like we have some guarantee. We’re certainly satisfied by this.” (Participant 102, village doctor)
- “Under the integrated management, we are responsible for various village clinics’ issues, including as finance, medical standards, equipment, and so on. Though integrated management is not yet conducted consistently, it shows positive trends.” (Participant 112, health manager)
- “Village doctors were quite free before, no one managed them. But now, THCs attempt to manage them as regular doctors, requiring them to work on time, keep medical records, participate in on-the-job training, and so forth. There is no doubt that this strategy will improve their work capacity comprehensively.” (Participant 117, health manager)

#### Professional reputation

Due to economic development, circumstances in China have changed greatly since the barefoot doctor era, particularly regarding the availability of health care. As such, many village doctors felt they were no longer in demand and obtained less social respect. Meanwhile, the doctor-patient relationship in sampled areas was just like the seller and buyer in a fair deal without emotional obligations. Thus, most village doctors tended to work in a fee-for-service manner with a lower sense of job satisfaction.

- “There were no doctors in my village before. If someone was sick, he/she had to travel far to find a doctor, even for those common diseases. I just wanted to change that situation, so I became a village doctor. But now, everything is different. There are many medicine sellers here. I am not in needed anymore. I am confused why I even chose to be a doctor.” (Participant 103, village doctor)
- “I prefer that [barefoot doctor] era. The living standards were almost the same among everyone. Local residents respected barefoot doctors a lot, since doctors and medicine were quite lacking at that time. But now, our villages don’t lack doctors or medicine anymore.” (Participant 209, village doctor)
- “In patients’ opinions, if they feel sick, you treat them, and they pay you back with money. They owe you nothing. It’s all that means, like a fair trade.” (Participant 201, village doctor)

## Discussion

The research findings show that job satisfaction of village doctors remains a crucial issue in Chinese rural health system, though the recent policies have some positive effect on it. In general, job satisfaction rate among sampled village doctors was 48.2%. Though it was still at a quite low level, it has improved notably compared to the result of another national survey on rural primary health workforce (5.4%) in 2011 [25]. Both the qualitative and quantitative results imply that those policies aimed at the village-level health workforce in recent years may have functioned positively as a whole.

The study used a mixed-method approach, which not only reveals the current status of job satisfaction of village doctors in China, but also illuminates the participants’ values, beliefs, perspectives and experiences in order to provide an explanation of this phenomenon. In this study, qualitative results triangulated quantitative results, allowing participants to provide the truth and explanations instead of researchers [26].

### Individual level factors

#### Age

We also found that age exhibited the most consistent positive correlation with job satisfaction. Senior village doctors were more likely to be satisfied, a result consistent with prior studies in China [27]. This may result from senior doctors having greater trust and respect from local residents [28], thus gaining more personal confidence and gratification. Similarly, high expectations among junior doctors could lead to lower satisfaction. Due to the relatively close nature of communities in rural areas, village doctors have to earn trust and slowly build long-term relationships with local residents. Thus, junior village doctors may not receive the respect they feel they deserve [29], particularly considering the erosion of the close doctor-patient relationship in rural areas and the increasing occurrence of medical disputes [30]. Compared to the older generation who became village doctors due to the government policies between 1960 and 1980, junior village doctors had many more options when planning their careers. Becoming a village doctor may not have matched their expectations, especially as they see their peers on other career paths receiving better incomes and occupation development [31]. As such, low job satisfaction among junior doctors may inevitably result long-term in a shortage in the rural health workforce.

### Income status

Village doctors with higher incomes are more likely to have a higher level of satisfaction, while lower and average income led to almost identical satisfaction, indicating that income is still an associated factor. Village doctors only receive a small subsidy for provision of public health services rather than a regular and reasonable, nationwide compensation plan. Instead, they must rely on fee-for-service activities, such as basic medical services and medicine sales, to generate income. Still, their incomes only fall approximately on par with the average of the village. This relatively low income status negatively affects village doctors’ personal views on the value of their work. As previous studies have claimed, providing a better compensation scheme for village doctors could improve such situation [32, 33]. However, this would likely be quite a large financial input of the government. More possible solutions for the long-term include creating a formal title for the village doctors to improve their reputation in society and enhancing the self-profitability of village doctors.

#### Pension plan

No significant difference was found between job satisfaction in connection to the pension plan, indicating that NRPP does not work as planned in terms of village doctors. As previous studies have suggested, a sufficient pension plan is critical for retention of village doctors in rural areas [34–38]. However, through various pilot programs [39, 40], NRPP has still not achieved the goal, mainly due to the low return rate [15]. As such, NRPP is limited to being a supplementary income for village doctors after retirement, rather than sufficient support [41]. Furthermore, NRPP is a general pension plan for all rural residents in China without distinctions between employed and unemployed. Since other medical workers in China are covered by urban basic endowment insurance system, which includes employees only, participants believed that only having NRPP available to village doctors undervalued their role, resulting in negligible or even negative effects on satisfaction.

#### Workload

Village doctors with high workloads are more likely to be unsatisfied with their job, as consistent with previous studies [42, 43]. Since 2009, village doctors have gradually been responsible for more basic public health services. This change increases their workload [14], as it is time- and energy- consuming to provide door-to-door services. Compared to a nation-wide survey, village doctors sampled in this study had even more workload than the average level of all community health workers in China [44]. In such a case, village doctors will inevitably feel stressed and exhausted, hindering the effective implementation of their work [45]. However, providing basic public health care services is essential since the Health Sector Reform. The amount of village doctors’ work on public health cannot be reduced, although it continues to add up and result in a negative effect on village doctors’ job satisfaction. Therefore, we suggest shifting the evaluation of village doctors’ performance from quantity-based to result-based.

#### Integrated management

Village doctors who work under high-level integrated management were more likely to be satisfied with their job, similar to results of a previous study [46]. Integrated management as a new policy implemented in rural health care recently, aims to include village doctors in the formal primary health care system under the supervision of THCs. Under this policy, village doctors get guidance and supervision from THCs for daily work, meanwhile, they receive financial and emotional support from THCs. According to Herzberg’s Two-Factor Theory of motivation, integrated management is meeting village doctors’ psychological and security needs and it motivates village doctors to work hard with higher job satisfaction. Working under THCs, they feel a stronger sense of camaraderie and belonging to part of the governmental healthcare system, instead of being private medical providers as past decades in China. This kind of social approval increases job satisfaction indirectly. Moreover, according to relative policies, THCs and county hospitals need to provide regular on-the-job trainings and clinical rotary opportunities for village doctors under high-level integrated management. As such, village doctors could receive advantageous knowledge and medical resources through continual contracts with upper level health organizations, substantially improving their professional skills and occupational prestige [47]. Thus, working under high-level integrated management makes village doctors more competitive in the local health market and therefore feel more satisfied.

### County level factors

Another significant determinant of satisfaction was the change in the medical landscape. With the Chinese economic reform and the resulting rapid urbanization, medical availability underwent rapid expansion, leaving behind the era of lacking doctors or medicine [48, 49]. As a result, rural residents are less dependent on village doctors, as basic medical services are available via local private pharmacies and clinics. Thus, the close patient-doctor relationship in rural areas has changed, diminishing village doctors’ sense of being needed [50]. Furthermore, during the “barefoot doctor” era, village doctors and local residents were not only doctor and patient, but also neighbors or even relatives [51]. Interpersonal relationships were quite stable back then, and emotional obligations were significant. However, with the rapid urbanization in rural China, local culture is disappearing, and traditional relationships get weakened [52]. As such, the doctor-patient relationship in rural areas has transformed gradually into a fair trade mechanism, reducing village doctors’ feeling of career fulfillment. Additionally, the expectation of village doctors may provide another explanation of this issue. During the “barefoot doctor” era, village doctors did not have more expectations on their work than as half-peasant-half-doctors supporting by limited and fixed income [12]. However, along with the economic development, most village doctors’ expectations for the job and income have increased. They expect higher income, on-the-job training, social respect, and so forth as being a village doctor. Village doctors, nowadays, are becoming more difficult to be satisfied in such case [14]. Thus, the more developed or modernized rural areas are, the lower the job satisfaction rate of village doctors.

There are several limitations in this study. First, although selected provinces are generally representative of the typical economic and health development characteristics in China, a larger geographical area would have more external validity. Secondly, the cross-sectional nature of the quantitative phase in this study dictates only correlation rather than causation in the relationships among variables. Finally, in the qualitative phase, participants who are willing to express their opinions are inevitably more likely to engage in interviews, which may lead to certain information bias. Meanwhile, there may be interviewer bias, since this study was conducted in three provinces and implemented by varied people. In the future, it would be useful to perform a more comprehensive study to further research on village doctors’ job satisfaction and how healthcare policies play a role.

## Conclusion

Job satisfaction of village doctors in China is still quite low, even though it has been improved since 2009, which implies the reform policies may have a positive trend as a whole. However, only one newly-formed policy, integrated management, is likely to improve village doctors’ job satisfaction. The others, basic public health services and new pension scheme, show negative functions. Thus, basic public health services and new pension scheme should be adjusted applicably, through mechanisms such as promoting the work efficiency of village doctors and improving the strength of village doctors’ pension plan. Moreover, these findings about the Chinese health care reform experience may also be relevant for other developing countries in maintaining community health workforce on a broader scale.

## Appendix 1

**Table 5:** Basic demographic information about interviewees

Num.	Participant	Age (year)	Gender	Current Position
1	101	20–39	male	health manager
2	102	60+	male	village doctor
3	103	40–59	female	village doctor
4	104	20–39	male	village doctor
5	105	60+	female	village doctor
6	106	40–59	female	health manager
7	107	60+	male	village doctor
8	108	20–39	female	health manager
9	109	20–39	male	village doctor
10	111	20–39	male	health manager
11	112	40–59	male	health manager
12	113	60+	male	village doctor
13	114	20–39	female	village doctor
14	115	40–59	male	village doctor
15	116	20–39	male	village doctor
16	117	40–59	female	health manager
17	118	20–39	male	health manager
18	201	40–59	male	village doctor
19	202	40–59	male	health manager
20	203	20–39	male	village doctor
21	204	40–59	male	village doctor
22	205	20–39	female	village doctor
23	206	20–39	male	village doctor
24	207	40–59	male	health manager
25	208	40–59	female	village doctor
26	209	40–59	male	village doctor
27	210	20–39	male	health manager
28	301	40–59	male	health manager
29	302	40–59	male	health manager
30	303	20–39	male	health manager
31	304	20–39	male	village doctor
32	305	60+	male	village doctor
33	306	20–39	male	village doctor
34	307	60+	male	village doctor

## Appendix 2

**Table 6:** Differences in individual-level characteristics of village doctors’ workload

Variables	Low workload (%)	High workload (%)	*P* value ^a^
Gender			0.005
Male	477 (51.57)	448 (48.43)	
Female	180 (60.81)	116 (39.19)	
Age (years)			0.752
20–39	189 (53.09)	167 (46.91)	
40–59	349 (53.45)	304 (46.55)	
60 +	119 (56.13)	93 (43.87)	
Education			<0.001
≤Junior high school	106 (69.28)	47 (30.72)	
Secondary school	450 (54.41)	377 (45.59)	
>Secondary school	101 (41.91)	140 (58.09)	
Average monthly income (CNY)			0.006
Low	244 (59.22)	168 (40.78)	
Ordinary	217 (54.11)	184 (45.89)	
High	196 (48.04)	212 (51.96)	
Pension plan			0.098
None	47 (59.49)	32 (40.51)	
NRPP	427 (51.69)	399 (48.31)	
Others	183 (57.91)	133 (42.09)

**Table 7:** Differences in individual-level characteristics of integrated management

Variables	Low integrated management (%)	High integrated management (%)	*P* value^a^
Gender			<0.001
Male	406 (43.89)	519 (56.11)	
Female	165 (55.74)	131 (44.26)	
Age (years)			0.892
20–39	168 (47.19)	188 (52.81)	
40–59	307 (47.01)	346 (52.99)	
60 +	96 (45.28)	116 (54.72)	
Education			<0.001
≤Junior high school	72 (47.06)	81 (52.94)	
Secondary school	356 (43.05)	471 (56.95)	
>Secondary school	143 (59.34)	98 (40.66)	
Average monthly income (CNY)			0.001
Low	217 (52.67)	195 (47.33)	
Ordinary	158 (39.40)	243 (60.60)	
High	196 (48.04)	212 (51.96)	
Pension plan			0.542
None	41 (51.90)	38 (48.10)	
NRPP	379 (45.88)	447 (54.12)	
Others	151 (47.78)	165 (52.22)	

## Abbreviations

CMS: Cooperative Medical Scheme
CNY: Chinese Yuan
GDP: Gross domestic product
ICC: Intra-class correlation coefficient
NRPP: New Rural Pension Program
ORs: Odds ratios
THCs: Township Health Centers.
